# A BRET-based assay reveals collagen–Hsp47 interaction dynamics in the endoplasmic reticulum and small-molecule inhibition of this interaction

**DOI:** 10.1074/jbc.RA119.010567

**Published:** 2019-09-06

**Authors:** Shinya Ito, Masazumi Saito, Masahito Yoshida, Koh Takeuchi, Takayuki Doi, Kazuhiro Nagata

**Affiliations:** ‡ Institute for Protein Dynamics, Kyoto Sangyo University, Kyoto 603-8555, Japan; § Graduate School of Pharmaceutical Sciences, Tohoku University, Sendai 980-8578, Japan; ¶ National Institute of Advanced Industrial Science and Technology, Tokyo 135-0064, Japan; ‖ Department of Molecular Biosciences, Faculty of Life Sciences, Kyoto Sangyo University, Kyoto 603-8555, Japan; ** CREST, Faculty of Life Sciences, Kyoto Sangyo University, Kyoto 603-8555, Japan

**Keywords:** molecular chaperone, collagen, heat shock protein (HSP), protein–protein interaction, bioluminescence resonance energy transfer (BRET), fibrosis, serpin, extracellular matrix, heat shock protein 47 (Hsp47), PPI inhibitor, SERPINH1

## Abstract

Molecular chaperones perform pivotal roles in proteostasis by engaging in protein–protein interactions (PPIs). The collagen-specific molecular chaperone Hsp47 (heat shock protein 47) interacts with procollagen in the endoplasmic reticulum (ER) and plays crucial roles in collagen synthesis. PPIs between Hsp47 and collagen could offer a therapeutic target for fibrosis, which is characterized by abnormal collagen accumulation in the extracellular matrix of fibrotic organs. Herein, we established a bioluminescence resonance energy transfer (BRET) system for assessing Hsp47–collagen interaction dynamics within the ER. After optimization and validation of the method, we could demonstrate inhibition of the interaction between Hsp47 and collagen by a small molecule (Col003) in the ER. Using the BRET system, we also found that Hsp47 interacts not only with the Gly-Pro-Arg motif but also weakly with Gly-Pro-Hyp motifs of triple-helical collagen in cells. Moreover, we found that the serpin loop of Hsp47 (SerpinH1) contributes to its binding to collagen. We propose that the method developed here can provide valuable information on PPIs between Hsp47 and collagen and on the effects of PPI inhibitors important for the management of fibrotic disorders.

## Introduction

Hsp47 (heat shock protein 47) is a collagen-specific molecular chaperone that localizes in the endoplasmic reticulum (ER)^3^ and plays a crucial role in collagen synthesis in vertebrates (1). Procollagen is co-translationally inserted into the ER and folded into a triple-helical structure. Hsp47 binds to and stabilizes the triple-helical portion of procollagen, stimulating triple helix formation by preventing unfolding, aggregation, and bundle formation. After Hsp47 dissociates from procollagen in the *cis*-Golgi in a pH-dependent manner, Hsp47 returns to the ER via the KDEL receptor. Knockout (KO) of *hsp47* results in embryonic lethality in mice caused by a lack of a basement membrane composed of type IV collagen (2). Procollagen secretion was delayed, and collagen accumulation in the extracellular matrix was decreased in mouse embryonic fibroblasts from *hsp47* KO mice (3). Although overexpression of WT Hsp47 recovered collagen accumulation in *hsp47* KO mouse embryonic fibroblasts, Y365A mutant Hsp47 lacking the ability to bind collagen cannot recover collagen production (4), suggesting that protein–protein interactions (PPIs) between Hsp47 and collagen are indispensable for collagen synthesis.

Fibrotic disease, characterized by abnormal collagen accumulation, impairs normal function in various organs including liver, lung, and kidney. An efficient treatment for the large number of patients suffering from fibrotic disease worldwide is not yet available. Inhibition of collagen synthesis is considered a potential therapeutic strategy for fibrotic disease (5). Although many drug candidates for fibrosis target signal transduction related to transcription of the collagen gene, collagen protein synthesis could also be targeted in fibrosis treatment. Knockdown of *Hsp47* expression by short hairpin RNA or siRNA can suppress the correct folding and accumulation of collagen, resulting in inhibition of liver fibrosis progression (6, 7). Thus, Hsp47 is considered a promising molecular target for fibrosis, and knockdown of *hsp47* is under phase II clinical trials for idiopathic pulmonary fibrosis. Previous studies revealed that PPIs between Hsp47 and collagen are indispensable for collagen synthesis (4). Thus, exploring small molecule compounds that inhibit Hsp47–collagen interactions could offer a beneficial therapeutic strategy for fibrosis treatment.

From comprehensive screening of small molecule compounds that inhibit the interaction of Hsp47 with collagen, we obtained compound Col003 that causes delayed procollagen secretion and inhibits collagen accumulation in the extracellular matrix (4). Col003 directly binds Hsp47 but not collagen and inhibits the Hsp47–collagen interaction *in vitro*. Based on NMR experiments, we found that Col003 competitively inhibits binding of Hsp47 to collagen because the binding sites for Col003 and collagen on Hsp47 are quite close and may overlap. Although secretion of collagen into the medium was inhibited in the presence of Col003, it remains unclear whether Col003 inhibits PPIs between Hsp47 and collagen in the ER.

Detection of PPIs between Hsp47 and collagen in the ER has been reported using a split GFP system and immunoprecipitation with a cross-linking agent (8, 9). Because of the irreversibility of these PPI detection methods, the inhibitory effects of small molecules cannot be correctly evaluated in cells. The bioluminescence resonance energy transfer (BRET) system could prove effective for evaluating PPIs within the cells. When the distance between Nano-luciferase (NLuc) as energy donor and Halotag fluorescent ligand as energy acceptor is small and the relative orientation of the two dipole moments is suitable, fluorescence emission from the acceptor can be observed. Because the Halotag ligand and substrate of NLuc are membrane-permeable, PPIs can be detected in living cells. BRET systems have been used to investigate interactions between MDM2 (mouse double minute 2) and tumor suppressor p53 in the cytosol and between G protein–coupled receptor proteins on the plasma membrane (10). However, PPIs within the ER have not been explored using this approach.

In the present study, we established a BRET-based detection system for Hsp47–collagen PPIs in the ER. We optimized the combinations and constructs and verified whether Col003 inhibits the interaction between collagen and Hsp47 within the ER.

## Results

### Establishment of the BRET system for detection of the Hsp47–collagen interaction in the ER

**Figure 1.**
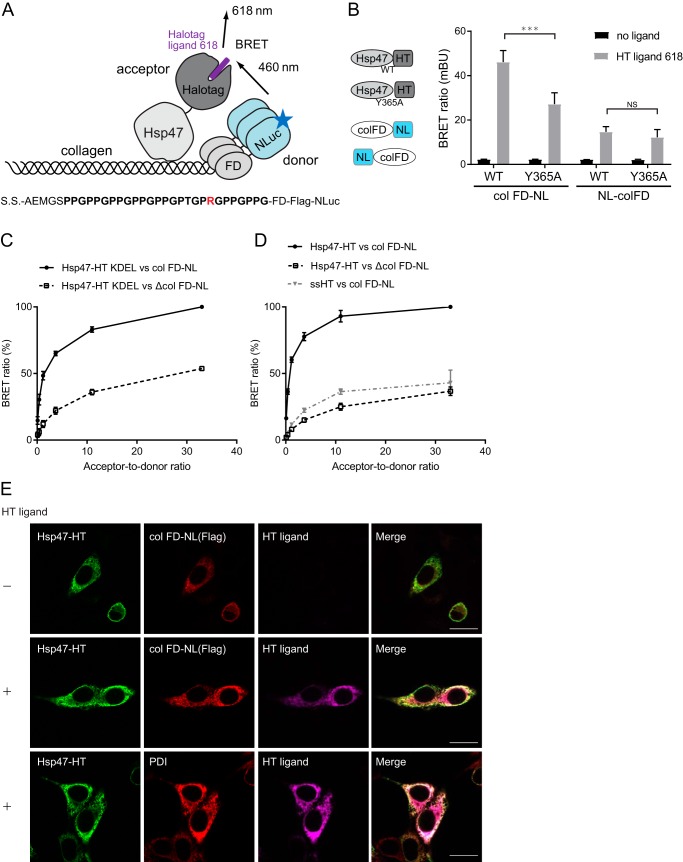
**Development of the BRET system for assessing Hsp47–collagen interactions in the ER.** *A*, schematic view of BRET analysis of the Hsp47–collagen interaction. The signal sequence (*S.S.*)–collagen peptide (amino acid sequence shown below) fused to the foldon (colFD) possessing NLuc as an energy donor interacts with Hsp47-HT/HT fluorescent ligand 618 as an energy acceptor. The key arginine residue is colored *red. B*, comparison of tag position on BRET efficiency. The Y365A mutant of Hsp47 displays reduced binding to collagen and was used as a negative control. NLuc (*NL*) was added to colFD at the N or C terminus. The BRET ratio was calculated from the signal at 618/460 nm (×1,000). The results are presented as means ± S.D. (*n* = 3, Student's *t* test). *NS*, not significant. *C*, donor saturation assay of colFD-NL/Hsp47-HT KDEL and ΔcolFD-NL/Hsp47-HT KDEL pairs. The results are presented as means ± S.D. of three independent experiments performed in duplicate. *D*, donor saturation assay of colFD-NL/Hsp47-HT, ΔcolFD-NL/Hsp47-HT, and colFD-NL/ssHT pairs. The maximum BRET ratio is shown as 100%, which was calculated by subtracting the value with no HT ligand. The results are presented as means ± S.D. of three independent experiments performed in duplicate. *E*, immunofluorescence of BRET pairs. The HT ligand can enter the ER. PDI was used as an ER marker. *Scale bar*, 20 μm.

BRET systems are used to detect PPIs in cells, particularly in the cytosol (10). Herein, to explore the interaction dynamics between Hsp47 and collagen in the ER, we employed a BRET system to investigate PPIs between Hsp47 and a collagen model peptide fused with the bacteriophage T4 fibritin foldon (FD) domain, which spontaneously forms a trimer and therefore stabilizes the triple-helical structure of collagen (11). The collagen model peptide consists of nine Pro-Pro-Gly collagen repeats containing the Thr-Gly-Pro-Arg sequence, the arginine of which reportedly enables the recognition of the collagen repeat by Hsp47 *in vitro* (12). Hsp47 also recognizes the amino acid in the Yaa^−3^ position in the sequence Yaa^−3^-Gly-Xaa-Arg (13). Specifically, Hsp47 most favors Thr and Pro at Yaa^−3^, followed by Ser, Hyp, Val, and Ala, but does not recognize Lys, Gln, or Glu (14). As shown in the schematic diagram in Fig. 1*A*, a BRET signal is presumably observed when Hsp47 harboring a Halotag fluorescent ligand is in close proximity to a collagen model peptide possessing an NLuc moiety.

We tested several combinations to establish a BRET system with the best energy transfer. Either NLuc (NL) or Halotag (HT) was added to the N or C terminus of Hsp47 and collagen (col) FD, respectively. A signal sequence for ER localization was added to the N terminus of each construct. As shown in Fig. S1*A*, Hsp47-NLuc and NLuc-Hsp47 expression resulted in partially cleaved proteins and insoluble products in HEK293 cells. Although Hsp47-HT was detected at the expected molecular size, the expression product of HT-Hsp47 was detected as a partially truncated protein (Fig. S1*A*). Thus, we selected pairs of Hsp47-HT and NL-colFD or colFD-NL (Fig. 1*B*). Because these constructs do not have ER retention sequences such as KDEL, they should be secreted into the culture medium. To detect PPIs between Hsp47-HT and colFD-NL within the ER, we washed cells with fresh medium and added NLuc substrate, a membrane-permeable reagent for measuring BRET. The BRET ratio (mBU) was calculated as the acceptor signal at 618 nm per donor signal (luminescence) at 460 nm (10). In the case of the Hsp47-HT and colFD-NL pair, a BRET signal was observed upon addition of HT ligand. The BRET signal of the Hsp47 Y365A mutant lacking the ability to bind collagen was lower than that of WT Hsp47, suggesting that this BRET signal depends on the interaction between Hsp47 and collagen (Fig. 1*B*). On the other hand, the BRET signal for the Hsp47-HT and NL-colFD pair was much smaller than that of the Hsp47-HT and colFD-NL pair, not different between WT and Y365A Hsp47, and almost the same as the background level in the ER. Based on these results, the Hsp47-HT and colFD-NL pair was adopted for further analysis of BRET.

The so-called donor saturation assay was next performed to estimate the specificity of BRET. When the amount of donor (colFD-NL) DNA used for transfection was held constant and the amount of acceptor (Hsp47-HT) DNA was gradually increased, a specific BRET signal increased in a hyperbolic manner and reached a plateau representing complete saturation of all donors with acceptor molecules (Fig. 1, *C* and *D*). By contrast, a BRET signal from nonspecific interactions resulting simply from close localization of two proteins should increase almost linearly with increasing amounts of acceptor. The Hsp47-HT KDEL and colFD-NL BRET pair yielded a much stronger signal than did the Hsp47-HT KDEL and ΔcolFD-NL (collagen deletion mutant of colFD-NL) pair at all ratios (Fig. 1*C*). The BRET pair consisting of Hsp47-HT without a KDEL retention signal and colFD-NL yielded a much stronger signal than the Hsp47-HT and ΔcolFD-NL pair, and it also exhibited donor saturation (Fig. 1*D*), indicating that the BRET signal between Hsp47-HT and colFD-NL was specific. The BRET signal for the ssHT and colFD-NL pair was only ∼20% by comparison, indicating nonspecific BRET interactions in the ER (Fig. 1*D*).

The localization of Hsp47-HT and colFD-NL within the ER generating a BRET signal was confirmed by immunofluorescence staining. Both Hsp47-HT and colFD-NL (containing a FLAG tag) co-localized and merged with each other, along with protein disulfide isomerase (PDI), an ER-resident protein (Fig. 1*E*). The signal from the membrane-permeable HT fluorescent ligand was captured by HT in the ER (Fig. 1*E*), suggesting that the BRET signal from Hsp47-HT and colFD-NL was indeed from the ER.

### Optimization of the BRET system for the Hsp47–collagen interaction in the ER

**Figure 2.**
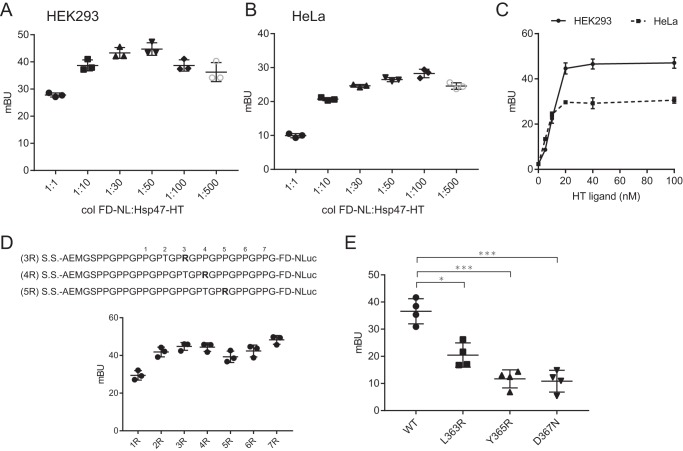
**Optimization and validation of the BRET system for evaluating Hsp47–collagen interactions in the ER.** *A* and *B*, BRET efficiency with different ratios of colFD-NL/Hsp47-HT pairs in HEK293 cells following transfection (*A*) and in HeLa cells (*B*). *C*, dependence of HT ligand concentration in HEK293 cells or HeLa cells. The results are presented as means ± S.D. (*n* = 3). *D*, the position of the key collagen arginine residue affects BRET efficiency. *E*, L363R, Y365R, and D367N mutants of Hsp47 lack the ability to bind collagen, resulting in reduced BRET efficiency (Student's *t* test; *n* = 4). *, *p* < 0.05; ***, *p* < 0.005.

To optimize BRET efficiency, the influence of the donor and acceptor ratio, the amount of HT ligand, and the position of the arginine residue were investigated. In HEK293 cells, the BRET signal was strongest at ∼40 mBU when the ratio of donor (colFD-NL) to acceptor (Hsp47-HT) was 1:50 (Fig. 2*A*). On the other hand, it was only ∼25 mBU at a ratio of 1:100 in HeLa cells (Fig. 2*B*). These differences between cell lines may reflect differences in the amount of endogenous Hsp47 and collagen in these cells (Fig. S1*B*). Regarding the amount of HT ligand, 20 nm HT ligand was sufficient for this BRET system with both cell lines (Fig. 2*C*). Because Hsp47 specifically recognizes and strongly binds to the arginine residue in the collagen triple helix, the position of arginine was altered from position 1R to 7R, and BRET analysis was performed. When the distance from the NLuc side was increased (1R), the BRET signal decreased (Fig. 2*D*), with a peak around the 3R to 4R position. Although the signal of the 7R construct was high, the position of arginine residue was too close to the foldon domain to analyze the correct interaction between Hsp47 and collagen triple helix. Thus, the 4R construct was used for the analysis.

Based on the co-crystal structure of Hsp47 and collagen model peptide, residues of Hsp47 responsible for interactions with collagen were assessed previously (13). Leu^363^, Tyr^365^, and Asp^367^ of Hsp47 are reportedly important for hydrophobic and hydrophilic interactions with collagen, respectively. Hsp47 mutants, in which these residues are altered, bind poorly to collagen in pulldown assays (13). These mutants displayed almost background level BRET signals (Fig. 2*E*), as shown for ssHT and ΔcolFD-NL in Fig. 1*D*, indicating the validity of this BRET system for probing the dynamic interactions of Hsp47 with collagen in the ER of living cells for the first time.

### Inhibition of the Hsp47–collagen interaction by a small molecule

**Figure 3.**
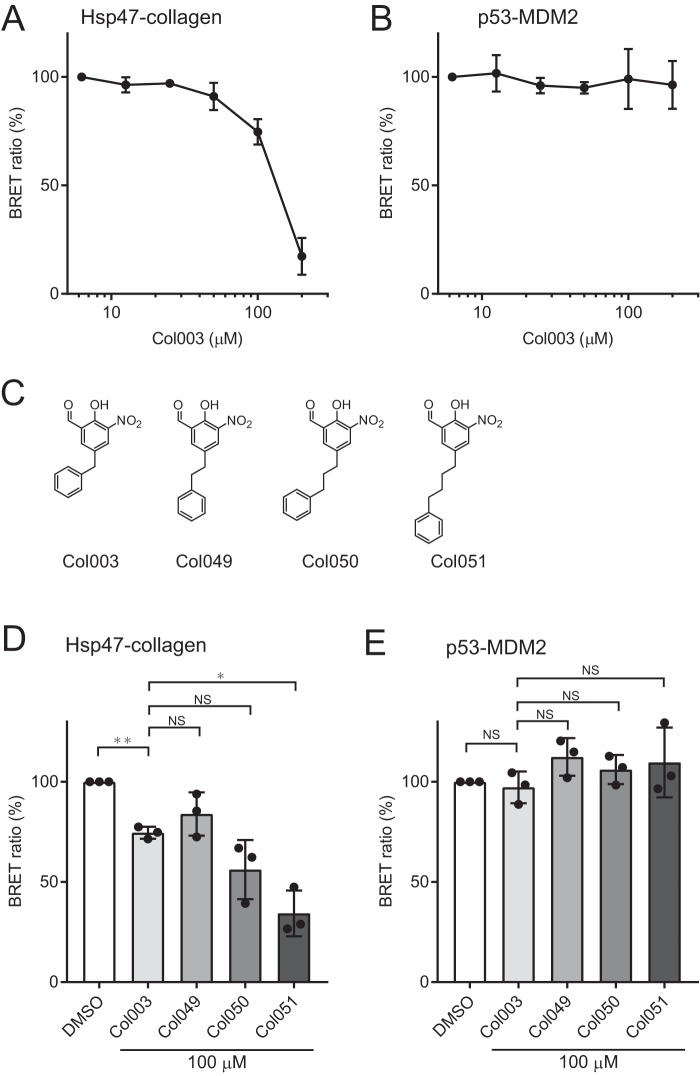
**The Hsp47–collagen interaction inhibitor Col003 reduces the BRET signal in the ER of living cells.** *A*, the BRET ratio (%) for Hsp47–collagen depends on the Col003 concentration. The results are presented as means ± S.D. (*n* = 3). *B*, the BRET ratio (%) of the control PPI pair p53-MDM2 is not dependent on the Col003 concentration. The results are presented as means ± S.D. (*n* = 3). *C*, structures of Col003 derivatives Col049, Col050, and Col051. Compounds differ in terms of the linker length between the two aromatic rings. *D*, BRET ratios (%) of Hsp47–collagen in the presence of Col003 derivatives. The BRET ratio with DMSO alone is shown as 100% (*n* = 3; Student's *t* test). *, *p* < 0.05; **, *p* < 0.01. *E*, BRET ratios (%) of p53-MDM2 in the presence of Col003 derivatives. The BRET ratio with DMSO alone is shown as 100% (*n* = 3; Student's *t* test). *NS*, not significant.

Inhibition of PPIs between Hsp47 and collagen is a therapeutic target for fibrosis treatment because Hsp47 mutants lacking the ability to bind to collagen cannot rescue the reduced accumulation of collagen in Hsp47 KO cells (4). We reported previously a small molecule (Col003) that binds Hsp47 and inhibits the PPI between Hsp47 and collagen *in vitro*. Using our BRET system, the *in vivo* inhibitory effects of Col003 on the Hsp47–collagen PPI in the ER were evaluated. Col003 reduced the BRET signal in a dose-dependent manner (Fig. 3*A*). By contrast, the BRET ratio (%) of the control p53-MDM2 PPI pair did not change in the presence of Col003 (Fig. 3*B*), suggesting that Col003 is a specific PPI inhibitor for Hsp47 in cells. To obtain more specific PPI inhibitors of Hsp47, we performed structure–activity relationship (SAR) analysis of Col003 derivatives Col049, Col050, and Col051 (Fig. 3*C*). These derivatives have a linker between the two aromatic rings differing in length from that in Col003. Interestingly, a longer linker in Col003 derivatives resulted in a more efficient inhibitory effect, which was again specific for the Hsp47–collagen PPI, but not for p53-MDM2 PPI (Fig. 3, *D* and *E*). This SAR information provides information that can help reduce fibrosis by identifying compounds that inhibit Hsp47–collagen interactions.

### Role of the arginine residue in the Hsp47–collagen interaction in the ER

**Figure 4.**
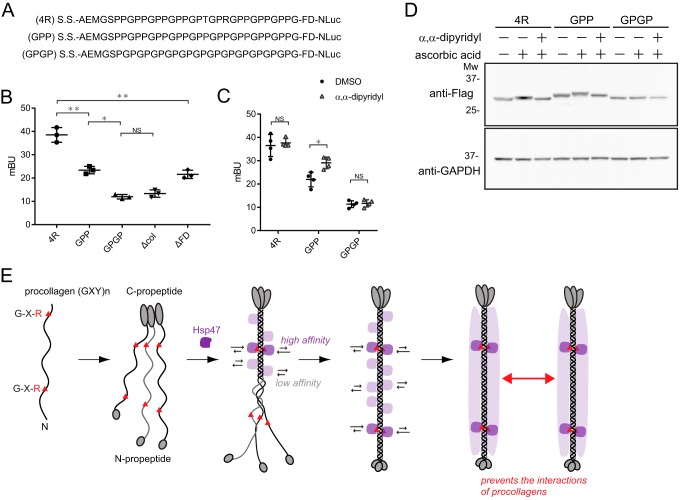
**The triple-helical structure of collagen affects the interaction with Hsp47.** *A*, the sequence used in this study. *S.S.*, signal sequence; *4R*, the position of the key collagen Arg from the C terminus. *B*, BRET ratio (mBU) for 4R, GPP, GPGP, Δcol, and ΔFD constructs (*n* = 3, Student's *t* test). *, *p* < 0.05; **, *p* < 0.01. *C*, α,α-dipyridyl treatment only affects the GPP construct. Collagen prolyl hydroxylase requires ascorbic acid and ferrous ions for activity. α,α-Dipyridyl is a ferrous ion chelator. *D*, immunoblotting analysis of *C*. Glyceraldehyde-3-phosphate dehydrogenase was used as a loading control. *Mw*, molecular weight. *E*, model of Hsp47 binding to procollagen in the ER. Hsp47 binds strongly to the Arg residue of the collagen triple-helical structure but also binds weakly to other sites on collagen. This “coating” of Hsp47 on procollagens could explain the collagen-specific molecular chaperone function of Hsp47.

Using this BRET system, we examined the interaction of Hsp47 with various molecules containing collagen-like sequences in the ER (Fig. 4*A*). The Gly-Pro repeat sequence, which does not form a triple-helical structure, gave the same BRET signal as that of a collagen domain-deleted construct, Δcol (Fig. 4*B*). When the proportion of triple-helical collagen to total collagen was decreased by deletion of the foldon domain (ΔFD), the BRET signal (and hence the Hsp47–collagen interaction) was decreased significantly. These results are consistent with those of *in vitro* studies using collagen model peptides and purified recombinant Hsp47 (rHsp47); rHsp47 binds only triple-helical collagen (12).

In the previous *in vitro* study, rHsp47 was shown to prefer arginine residues in the third position of (Gly-Xaa-Yaa) of collagen repeats (Gly-Pro-Arg) in the triple helix, but not hydroxyproline at the same position (Gly-Pro-Hyp) (12). Almost all prolines at the Yaa position are hydroxylated in the ER by prolyl hydroxylase in the monomeric form, and Hsp47 is thought to bind only to arginine localization sites on triple-helical procollagen in the ER. Meanwhile, GPP, an arginine-null collagen construct (Fig. 4*A*), reduced the BRET signal significantly, but interestingly, Hsp47 interacts with GPP more strongly than with GPGP and the Δcol construct (Fig. 4*B*), implying that Hsp47 may interact weakly with the Gly-Pro-Hyp motif of collagen in the ER. Prolyl 4-hydroxylase requires ascorbic acid and ferrous ions for hydroxylation activity (15). Treatment with α,α-dipyridyl, a ferrous ion chelator, affects the binding of Hsp47 to the GPP construct but not to 4R or GPGP constructs (Fig. 4*C*), consistent with the *in vitro* results; Hsp47 binds (Gly-Pro-Pro) more strongly than (Gly-Pro-Hyp). Hydroxylation of collagen peptides was confirmed by immunoblotting of these constructs with or without treatment with α,α-dipyridyl (Fig. 4*D*).

Using our BRET system, we found that Hsp47 binds weakly to arginine-null collagen, representing a novel finding for PPIs between Hsp47 and collagen in the ER. Based on the results of *in vivo* binding studies using the BRET system, we propose a model for binding between Hsp47 and procollagen in the ER (Fig. 4*E*). Hsp47 binds strongly to the Arg residue in the collagen triple-helical structure but also weakly to other Gly-Pro-Pro sites in the collagen triple helix. This “coating” of Hsp47 on procollagens may contribute to preventing unfavorable bundle formation or local unfolding of procollagens in the ER, revealing a possible function for Hsp47 as a collagen-specific molecular chaperone.

### Role of the serpin loop of Hsp47 on the Hsp47–collagen interaction in the ER

**Figure 5.**
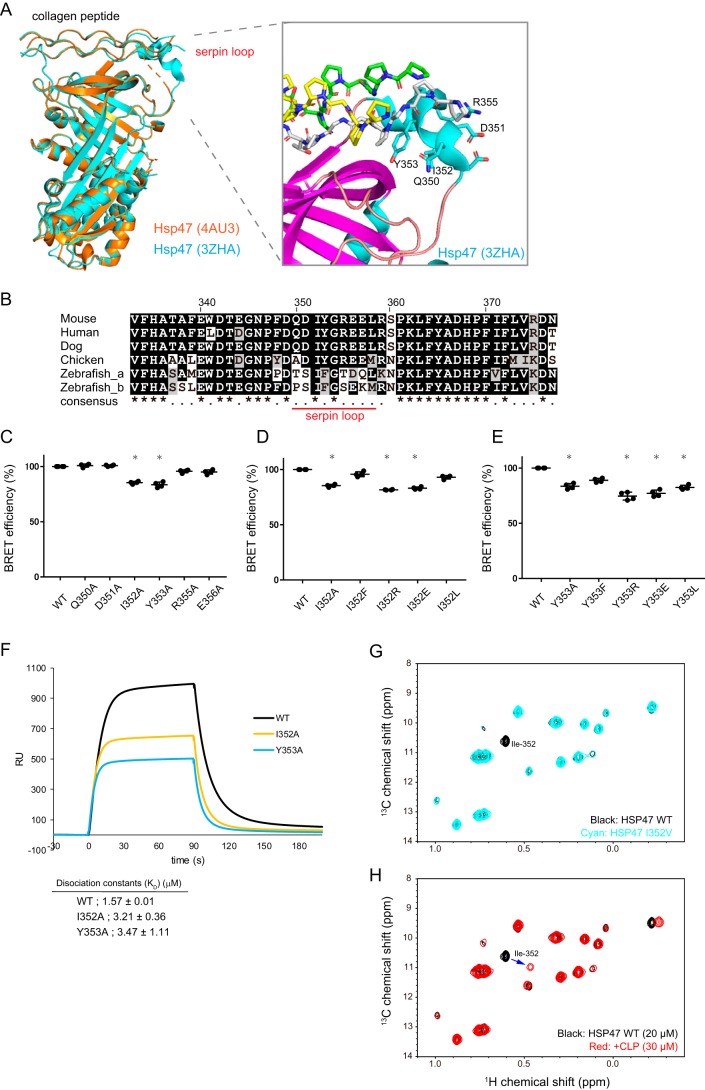
**The role of the serpin loop in the Hsp47–collagen interaction.** *A*, *left panel*, overview of the X-ray structures of Hsp47 (PDB codes 4AU2 and 3ZHA) in complex with collagen model peptide (*CLP*). *Right panel*, detailed view of the key residues in the serpin loop region of Hsp47 (stick model) that may interact with CLP (stick model with *yellow*, *white*, and *green* carbon atoms). The number of each amino acid was designated according to its position in the mouse Hsp47 sequence. *B*, sequence alignments of human, mouse, canine, and chick Hsp47. Clustal Omega software was used to make the alignments. The regions of the loop are marked by a *red line*. Ile^352^ and Tyr^353^ are in *bold. C–E*, the BRET ratio (%) for the interaction of Hsp47 WT and mutants with collagen in the ER. The results are presented as means ± S.D. (*n* = 4; Student's *t* test compared with WT). *, *p* < 0.05; **, *p* < 0.01. *F*, SPR analyses of the binding of recombinant mouse Hsp47 (rmHsp47) WT, I352A, and Y353A to collagen, which was immobilized to the sensor tip. The values of the dissociation constant (*K_D_*) for Hsp47–collagen were determined based on the those of a previous study (29). The data are represented as the means ± standard deviation of *n* = 3 replicates. *RU*, response units. *G*, titration of CLP with [U-^2^H,^1^H,^13^C-methyl Ile δ1] chick Hsp47. The Ile δ1 methyl regions of the ^1^H-^13^C HMQC spectra of the WT Hsp47 (*black*) and I352V mutant (*cyan*) are compared. The numbering is based on mouse Hsp47. *H*, comparison of the Ile δ1 methyl regions from the ^1^H-^13^C HMQC spectra of Hsp47 in the absence (*black*) and the presence of 30 μm CLP (*red*).

Hsp47 (SerpinH1) belongs to the serine protease inhibitor (serpin) superfamily (16), which possesses a distinctive loop region in a typical serpin structure. Some serpin family proteins inhibit proteases via a unique mechanism termed the suicide system, which involves a unique conformational change (17). When the target protease cleaves the reactive center loop (serpin loop) of a serpin protein acting as a protease substrate, an acyl bond is formed between the Ser in the loop and the protease. Then the loop is inserted into the serpin structure as a new β-sheet, which traps and inactivates the protease. Hsp47 is reported not to have this protease inhibitor function, suggesting that the serpin loop of Hsp47 has another function other than that of protease inhibition. Among the four crystal structures of Hsp47 deposited in the Protein Data Bank (PDB), the serpin loop of Hsp47 has not been solved in three PDB files (4AU2, 4AU3, and 4AU4). However, the loop region of Hsp47 is reported to have a helix-like structure in one PDB file (3ZHA), and this helix is located close to the collagen peptide (Fig. 5*A*). Loop region sequences are highly conserved among vertebrates, especially in mammals (Fig. 5*B*). Based on the Hsp47 structure, the tyrosine residue (Tyr^353^) in the loop region appears to engage in a hydrophobic interaction with collagen peptides (Fig. 5*A*).

Because we established a BRET system for monitoring the PPI between Hsp47 and collagen in the cell, we decided to use it to examine whether the serpin loop of Hsp47 is involved in binding to collagen. After introducing several mutations into the loop region of Hsp47-HT by alanine scanning (see *red line* in Fig. 5*B*), we examined the expression levels of these mutants in HEK293 cells by Western blotting, which confirmed that they were expressed at similar levels to the WT (Fig. S2, *A–C*). The BRET efficiencies of I352A and Y353A mutants were reduced slightly but significantly (Fig. 5*C*), suggesting that these two residues are involved in the interaction with collagen. Next, we changed these two amino acids to Phe, Arg, Glu, or Leu to characterize further their role in the interaction. BRET results showed that Ile^352^ could be replaced by Phe and Leu, whereas Tyr^353^ could only be replaced by Phe (Fig. 5, *D* and *E*). This result suggested that a hydrophobic residue and an aromatic ring at positions Ile^352^ and Tyr^353^, are important for the interaction, respectively. To verify the role of these two residues more precisely, we purified recombinant Hsp47 I352A and Y353A mutants and determined their affinities for collagen using surface plasmon resonance (SPR). Compared with WT (*K_d_*, 1.5 μm), I352A and Y353A mutants had lower affinities (I352A, *K_d_* = 3.3 μm; Y353A, *K_d_* = 3.6 μm) (Fig. 5*F* and Fig. S3*A*). The dissociation rates of these two mutants were faster than that of the WT (Fig. S3*B*).

Finally, we confirmed the importance of Ile^352^ in the serpin loop for the interaction with collagen by performing NMR spectra analysis in the presence and absence of a collagen model peptide (CLP), (GPP)_10_. Ile residues were isotopically labeled with NMR-active ^13^C nuclei in their δ1 methyl positions. ^13^C labeling allows selective detection of Hsp47 Ile resonances in the ^1^H-^13^C HMQC spectra (Fig. 5*G*). Compared with the WT spectrum, the I352V mutant spectrum revealed the absence of one signal, suggesting that this signal originated from Ile^352^. When CLP was titrated with Hsp47, the NMR signal of Ile^352^ clearly shifted from its original position (Fig. 5*H*), indicating that Ile^352^ is involved in the interaction with collagen. Combining the above results, including BRET, SPR, and NMR, we concluded that the serpin loop of Hsp47 is involved in the interaction with collagen via its Ile^352^ and Tyr^353^ residues.

## Discussion

PPIs play a crucial role in the regulation of various cellular functions including proteostasis and therefore provide a therapeutic target for many diseases (18). Indeed, PPI inhibitors are under study by numerous pharmaceutical companies (19). For example, tirofiban (Aggrastat), a small molecule inhibiting PPIs between fibrinogen and platelet integrin receptor glycoprotein IIb/IIIa (20), prevents platelet aggregation and has been approved for the prevention of blood clotting. Idasanutlin, an inhibitor of the interaction between MDM2 and p53, is under phase III clinical trials for acute myeloid leukemia (21). Many PPI inhibitors have been developed, but relatively few have reached the market.

In general, high-throughput screening of PPI inhibitors is performed using a combination of biophysical methods, including time-resolved FRET and α-screening, and hit compounds are further evaluated by SPR, isothermal titration calorimetry, and other methods before cellular responses are examined. One of the difficulties in developing PPI inhibitors is that there are gaps in PPIs *in vitro* and PPIs *in vivo* because they tend to be relatively large molecules, which reduces their cell membrane permeability (22). Considering these difficulties, PPI detection in cells could provide a way to relate the results of *in vitro* drug screening with cellular responses to these drugs.

To this end, we herein established a BRET system for detecting Hsp47–collagen interactions in the ER. Mutation of several residues on the collagen interaction surface of Hsp47 reduced the BRET signal. A small molecule compound, Col003, inhibits Hsp47–collagen interactions within the ER. In our previous study, we succeeded in detecting interactions between procollagen and Hsp47 by adopting a split GFP system (9). However, this system could not detect the dissociation of Hsp47 from procollagen because fragments of GFP cannot dissociate once they associate to form mature GFP. Herein, for the first time, we succeeded in detecting the *in vivo* binding of Hsp47 to a collagen peptide within the ER using this BRET system. This technique is thought to be useful for evaluating compounds that inhibit PPIs in the ER, where many therapeutic PPI targets such as secretory proteins and membrane proteins are synthesized (23).

Molecular chaperones including Hsp70, Hsp90, and Hsp60 play essential roles in proteostasis via protein folding, preventing protein aggregation and degradation. Their roles depend on PPIs between chaperones and their substrates. PPI inhibitors of molecular chaperones are also therapeutic targets because they may regulate the synthesis of substrate proteins and modulate proteostasis (24). Hsp47, a collagen-specific molecular chaperone, is essential for collagen folding in the ER. PPIs between Hsp47 and collagen are required for collagen synthesis, representing a therapeutic target for fibrosis caused by abnormal collagen accumulation. Arginine residues in collagen are known to be important for binding to Hsp47, but we found that Hsp47 also binds weakly to arginine-null collagen in the ER using our BRET system (Fig. 4*C*), suggesting that Hsp47 may have more binding sites on triple-helical collagen. This could indicate another important role for Hsp47 as a chaperone; Hsp47 may more widely cover the entire portion of procollagen in the ER to prevent the inappropriate bundling among collagen triple helices because of the high surface hydrophobicity of procollagen triple helices. In Hsp47 KO cells, procollagen forms aggregates or bundles in the ER (25), suggesting the importance of this “weak coating” of procollagen by Hsp47 in the prevention of aggregation.

Furthermore, to demonstrate the utility of this BRET system, we used the BRET assay to elucidate the mechanism of the interaction between Hsp47 and collagen. Hsp47 belongs to the serpin family, in which the serpin loop plays a crucial role in serpin function. However, the function of the serpin loop of Hsp47 has not been previously reported. Using the BRET method, we found that the loop region of Hsp47 contributes to its binding to collagen (Fig. 5). It is worth noting that when the collagen peptide Ac-PPGPPGPPGPRGPPGPPG-NH_2_ was used for co-crystallization, the serpin loop could not be solved (PDB code 4AU3), but when the collagen peptide Ac-PPGPPGPTGPRGPPGPPG-NH_2_ was used, the loop was shown to adopt a helix structure (PDB code 3ZHA), whereas the effect of the TGPR sequence in the latter collagen peptide on the stability of the loop region was unclear. The collagen sequence used in BRET was (PPG)_4_PTGPRG(PPG)_3_, in which the proline residues in the *Y* position of G*XY* repeats are hydroxylated (Fig. 4). It should be noted that amino acid sequence in the *XY* position of (Gly-Xaa-Yaa) show considerable diversity among endogenous procollagens; thus, the contribution of the Ile and Tyr resides in the loop region to the interaction with collagen might differ depending on which amino acids are present in the *XY* positions of collagen. For example, the phenolic hydroxy group of tyrosine may form a hydrogen bond with a side chain of an amino acid at the *XY* positions. Although the involvement of the serpin loop in the interaction with collagen was established for the first time in this report, the importance of the serpin loop on binding to collagen needs further investigation.

Information on the compound binding site on Hsp47 could be obtained from SAR analysis of Col003 derivatives. Derivative Col051 possessing a longer linker between the two aromatic groups more effectively inhibited Hsp47–collagen interactions in the ER than Col003. Interestingly, the longer the linker (shorter to longer in Col049, Col050, and Col051), the more effective the inhibitory effect. There are various explanations for these SAR observations: the phenyl group may acquire the ability to more strongly interact with Hsp47, the alkyl linker between the two aromatic rings may engage in hydrophobic interactions with Hsp47, and the phenyl group may overlap the binding site for collagen more effectively, increasing competitive inhibition. A co-crystal structure of Hsp47 and Col003 derivatives and/or NMR experiments could provide more information on binding.

There are several technical complications when using BRET systems in the ER. First, if one or both members of the BRET pair are secreted, the donor–acceptor ratio will change during the secretion process, making it necessary to measure BRET after thorough washing of secreted proteins into the medium. Second, BRET in the ER suffers from a high background compared with that in the cytosol (10), probably because the protein concentration within the ER is higher than in the cytosol (26), causing nonspecific interactions among proteins. Despite these technical difficulties, BRET could prove indispensable for evaluating PPIs in the cells, particularly in the ER. Using this tool, we probed the Hsp47–collagen interaction dynamics within the ER for the first time and demonstrated that Col003 works as a PPI inhibitor of Hsp47–collagen *in vivo*. We have therefore developed a method to assess PPIs *in vivo* and laid the basis for performing SAR analysis of Col003 derivatives, which could prove useful for screening small molecules as potential therapeutic treatments for fibrotic diseases.

## Experimental procedures

### Compound synthesis

The Col003 compound was synthesized as previously reported (4). The synthesis of Col049, Col050, and Col051 is described in the study (27), where Col049, Col050, and Col051 refers to 5eE, 5eF, and 5eG, respectively.

### Plasmids and antibodies

The colFD was synthesized artificially with NheI/EcoRI sites, the DNA sequence GCTAGCATTGCCACCATGCGCTCACTGCTGCTCCTGTCCGCCTTCTGCCTCCTTGAGGCTGCCTTGGCTGCGGAAATGGGGAGTCCGCCCGGTCCTCCAGGACCACCTGGCCCTCCCGGACCTACAGGCCCCAGAGGGCCACCGGGACCTCCAGGTCCACCCGGCTCTGGCTACATTCCCGAAGCACCCAGGGATGGGCAGGCCTATGTCCGGAAAGATGGCGAGTGGGTGCTGCTGAGCACCTTCCTGATCGACTACAAGGACGATGACGACAAGGAGTTTGAATTC, and the amino acid sequence MRSLLLLSAFCLLEAALAAEMGSPPGPPGPPGPPGPTGPRGPPGPPGPPGSGYIPEAPRDGQAYVRKDGEWVLLSTFLIDYKDDDDKEFEF. The colFD sequence was inserted into the pNLF1-N, pNLF1-C, pHTN-HaloTag (HT), and pHTC-HT (Promega, Madison, WI) vectors. Where needed, a signal sequence was added to the N terminus, and the reading frame was adjusted accordingly. Other variants of colFD constructs were created as described above, for which amino acid sequences are shown in figures. The mouse Hsp47 gene was inserted into the EcoRI site of the pNLF1-N, pNLF1-C, pHTN-HT, and pHTC-HT vectors, and signal sequences and/or reading frames were adjusted accordingly. Hsp47 mutants including L363R, Y365A, Y365R, and D367N were generated using primers and PrimeSTAR Max DNA polymerase (catalog no. R045A; Takara Bio, Shiga, Japan). All sequences were confirmed using a Genetic Analyzer 3130 (Applied Biosystems, Waltham, MA). The PPI control pair (MDM2-NanoLuc and p53-HaloTag) was purchased from Promega.

Hsp47 inhibitory compound Col003 and its derivatives were synthesized and verified in terms of chemical structure as described in a previous study (4). The following antibodies were used for immunoblotting: monoclonal anti-FLAG M2 antibody (catalog no. F3608; Sigma–Aldrich), monoclonal mouse anti–glyceraldehyde-3-phosphate dehydrogenase (catalog no. 5G4-6C5; HyTest, Turku, Finland), monoclonal anti-Hsp47 antibody (M16. 10A1; Enzo Life Sciences, Inc.), polyclonal anti-Halotag antibody (catalog no. G928A; Promega), and monoclonal anti-collagen type I antibody (catalog no. SAB1402151, clone 3G3; Sigma–Aldrich).

### BRET assay

The BRET assay was performed using reagents from Promega (catalog no. N1661) as described previously (10). In brief, HEK293 or HeLa cells were cultured in Dulbecco's modified Eagle's medium (high glucose) with 10% fetal bovine serum (FBS) and penicillin/streptomycin (Nacalai Tesque, Kyoto, Japan). The cells were transiently transfected with BRET constructs Hsp47-HT and colFD-NL using Lipofectamine 2000 (catalog no. 11668027; Thermo Fisher) according to the manufacturer's instructions. After 5 h, the cells were replated into a white 96-well poly-d-lysine–coated culture plate (catalog no. 152028; Thermo Fisher) at 3.6 × 10^4^ cells/well (HEK293 cells) or 2.25 × 10^4^ cells/well (HeLa cells) in 90 μl of Opti-MEM medium containing 4% FBS and 200 μm l-ascorbic acid phosphate magnesium salt n-hydrate (catalog no. 013-19641; FUJIFILM Wako Pure Chemical Corporation, Osaka, Japan). Transfected cells were labeled using the 20–100 nm HT ligand 618 (catalog no. G9801; Promega). After incubation for 19–21 h at 37 °C, the growth medium was replaced by 50 μl of medium consisting of Opti-MEM with 4% FBS and NanoBRET substrate furimazine (catalog no. N1571; Promega; 1/2,000 dilution). After a 10-min incubation at 37 °C, the BRET signal was measured at 37 °C using a Varioskan LUX plate reader (Thermo Fisher) using a 460-nm HBW80 filter for the donor signal and a 610-nm long-pass filter for the acceptor signal. For evaluating Hsp47 inhibitors, after incubation for 19–21 h following replating, transfected cells were treated with compounds at the indicated concentrations for 2 h. After washing three times with Opti-MEM, the medium was replaced with 50 μl of Opti-MEM with 4% FBS and BRET substrate, and the BRET signal was detected as described above.

### Immunofluorescence experiments

HEK293 cells were transfected with 300 ng of colFD-NL, 300 ng of Hsp47-HT, or 400 ng of empty vector; incubated with HT ligand for the same time as in the BRET assays; and fixed with 4% (w/v) paraformaldehyde for 15 min. After washing three times with PBS, the fixed cells were permeabilized using 0.1% Triton X-100 in PBS for 5 min and blocked with PBS containing 2% goat serum and 10% glycerol for 50 min at room temperature. Rabbit polyclonal antibodies recognizing Halotag (catalog no. G9281; Promega; 1/500 dilution), monoclonal anti-FLAG M2 antibody (catalog no. F1804; Sigma–Aldrich; 1/400 dilution), and monoclonal anti-PDI antibody (catalog no. ADI-SPA-891; Enzo Life Sciences, NY; 1/400 dilution) were reacted as primary antibodies for 1 h at room temperature. After washing three times with PBS, Alexa Fluor 488–conjugated anti-rabbit IgG (catalog no. A11034, Thermo Fisher; 1/400 dilution) and Alexa Fluor 555-conjugated anti-mouse IgG (catalog no. A21424; Thermo Fisher; 1/500 dilution) were reacted as secondary antibodies for 1 h at room temperature. After mounting with ProLong Gold antifade mountant (catalog no. P10144; Thermo Fisher), fluorescent signals were analyzed using an LSM 700 confocal fluorescence microscope (Carl Zeiss) with an appropriate setup of lasers, beam splitters, and filters for Alexa Fluor 488, Alexa Fluor 555, and HT ligand 618.

### Surface plasmon resonance

SPR (Biacore T200; GE Healthcare) was used to determine the affinity of recombinant mouse Hsp47 (rmHsp47) for collagen. Collagen (pig's skin type I-C (Nitta Gelatin Inc.)) was covalently immobilized on flow cell 2 in a Biacore CM5 sensor chip via the standard amine coupling reaction. The final response was ∼2,000 response units, where 1 response unit corresponds to 1 pg of protein per mm^2^. Flow cell 1 was coated with ethanolamine and used as reference spots. RmHsp47 was purified as described previously (28). The running buffer was HBS-P + running buffer (10 mm HEPES, 0.15 m NaCl, and 0.05% surfactant P20, pH 7.4), and the flow cell temperature was maintained at 25 °C. RmHsp47 was injected separately into flow cell 1 and 2 with a contact time of 90 s and a dissociation time of 120 s at a flow rate of 10 μl/min. 10 mm HCl was injected for 60 s to remove bound rmHsp47 from collagen on the sensor chips. Data analysis for calculating the affinity was performed using the *k*_obs_ linearization method with Anabel software (29).

### NMR spectroscopy

The recombinant chicken Hsp47 was purified as described previously (4). All experiments were performed on Bruker Avance 800 MHz spectrometers equipped with cryogenic probes. All spectra were collected using a buffer containing 20 mm HEPES-NaOH (pH 7.4), 100 mm NaCl, 5% (v/v) deuterated glycerol, and 100% D_2_O at 298 K. The concentration of [U-^2^H, ^1^H^13^C-methyl Ile δ1] Hsp47 was set to 20 μm. Collagen model peptide (CLP) ((Pro-Pro-Gly)_10_·xH_2_O; Peptide Institute Inc.) was suspended in the same buffer at a trimeric concentration of 1 mm and directly added to the recombinant chicken Hsp47 solution at a trimeric concentration of 30 μm. Spectra were processed using TOPSPIN (Bruker Biospin) and analyzed with Sparky (30). Each spectrum was recorded for 3 h.

## Author contributions

S. I. and K. N. conceptualization; S. I., M. S., and K. T. investigation; S. I., M. Y., K. T., and T. D. methodology; S. I. writing-original draft; S. I. and K. N. writing-review and editing; M. S., M. Y., and T. D. resources; K. T. software; T. D. and K. N. supervision; K. N. funding acquisition.

## Supplementary Material

Supporting Information

## Footnotes

3 The abbreviations used are:

ER: endoplasmic reticulum
BRET: bioluminescence resonance energy transfer
PPI: protein–protein interaction
serpin: serine protease inhibitor
KO: knockout
PDB: Protein Data Bank
NLuc or NL: Nano-luciferase
FD: foldon
HT: Halotag
col: collagen
PDI: protein disulfide isomerase
SAR: structure–activity relationship
SPR: surface plasmon resonance
FBS: fetal bovine serum
CLP: collagen model peptide
colFD: collagen foldon
mBU: milli-BRET units
HMQC: heteronuclear multiple quantum correlation.
