# Supplementary material for: A BRET-based assay reveals collagen–Hsp47 interaction dynamics in the endoplasmic reticulum and small-molecule inhibition of this interaction
Source: J Biol Chem. 2019 Sep 6;294(44):15962–72. doi: 10.1074/jbc.RA119.010567 (PMC6827286; doi:10.1074/jbc.RA119.010567)
Supplement: Supporting Information [file supp_294_44_15962__index.html]

A BRET-based assay reveals collagen–Hsp47 interaction dynamics in the endoplasmic reticulum and small-molecule inhibition of this interaction — Collagen–Hsp47 interaction dynamics in the ER — A BRET-based assay reveals collagen–Hsp47 interaction dynamics in the endoplasmic reticulum and small-molecule inhibition of this interaction — Collagen–Hsp47 interaction dynamics in the ER — Supporting Information 

# A BRET-based assay reveals collagen–Hsp47 interaction dynamics in the endoplasmic reticulum and small-molecule inhibition of this interaction

## Supporting Information

- Supporting Information - Supporting Information including Figure S1, S2 and S3
