# Supplementary material for: A BRET-based assay reveals collagen–Hsp47 interaction dynamics in the endoplasmic reticulum and small-molecule inhibition of this interaction
Source: J Biol Chem. 2019 Sep 6;294(44):15962–72. doi: 10.1074/jbc.RA119.010567 (PMC6827286; doi:10.1074/jbc.RA119.010567)
Supplement: Supporting Information [file supp_RA119.010567_155084_1_supp_387482_px1fby.pdf]

Supporting Information

A BRET-based assay reveals collagen–Hsp47 interaction dynamics in the endoplasmic reticulum and small-molecule inhibition of this interaction

Shinya Ito[1], Masazumi Saito[2], Masahito Yoshida[2, §], Koh Takeuchi[3], Takayuki Doi[2], and Kazuhiro Nagata[1,4,5, *]

Running title: Collagen–Hsp47 interaction dynamics in the ER

*To whom correspondence should be addressed: Kazuhiro Nagata: Department of Molecular Biosciences, Faculty of Life Sciences, Kyoto Sangyo University, Kyoto 603-8555, Japan; nagata@cc.kyoto-su.ac.jp; Tel.: +81-75-705-3134; Fax: +81-75-705-3121

Keywords: molecular chaperone, collagen, heat shock protein 47 (Hsp47), fibrosis, protein-protein interaction, PPI inhibitor, bioluminescence resonance energy transfer (BRET), serpin, extracellular matrix, SERPINH1

Table of content

Figure S1. Immunoblot (IB) of Hsp47 constructs, endogenous Hsp47, and endogenous procollagen type I

Figure S2. IB of the Hsp47 serpin loop mutants

Figure S3. Sensor grams of surface plasmon resonance (SPR) detecting the binding of Hsp47 to collagen

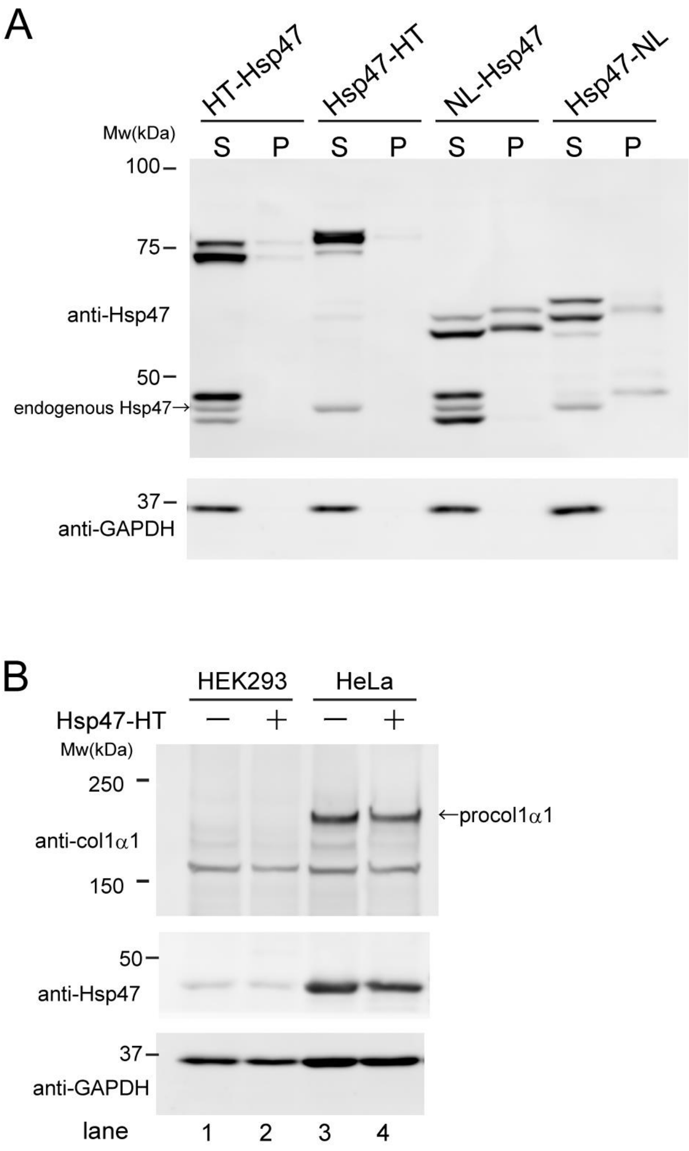

Figure S1. Immunoblot (IB) of Hsp47 constructs, endogenous Hsp47, and endogenous procollagen type I
(A) IB of extracts of HEK293 cells transfected with the Hsp47 constructs. HT, Halo-tag; NL, Nano-luciferase. After centrifugation of the cell lysate with a lysis buffer containing 1% NP-40, the supernatant fraction (S) and pellet fraction (P) were applied to each lane. Estimated molecular weights are HT-Hsp47, 75kDa; Hsp47-HT, 78kDa; NL-Hsp47, 65kDa; Hsp47-NL, 68kDa. GAPDH was used as a loading control. Mw, molecular weight. (B) The difference in the expression level of endogenous Hsp47 and procollagen in HEK293 cells and HeLa cells. GAPDH was used as a loading control. Mw, molecular weight.

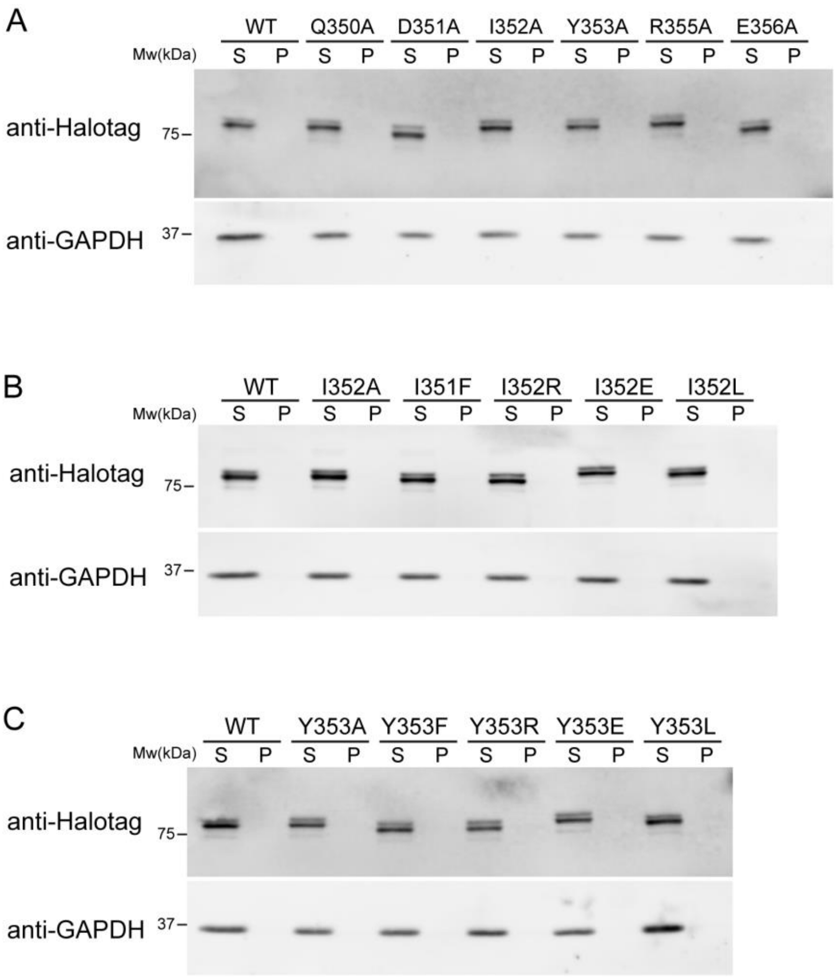

Figure S2. Immunoblot of the serpin loop mutants of Hsp47-Halotag (HT)

(A) The expression level of Hsp47-HT mutants used in Fig. 5C. After centrifugation of the cell lysate with a lysis buffer containing 1% NP-40, a supernatant fraction (S) and pellet fraction (P) were loaded into each lane. The expression levels of the serpin loop mutants were the same as that of wild type. Notably, no bands were detected in the pellet fraction, suggesting that the mutation did not cause instability of Hsp47 like Hsp47 OI mutants. GAPDH was used as a loading control. (B) IB of Ile352 mutants of Hsp47-HT (C) IB of Tyr353 mutants of Hsp47-HT

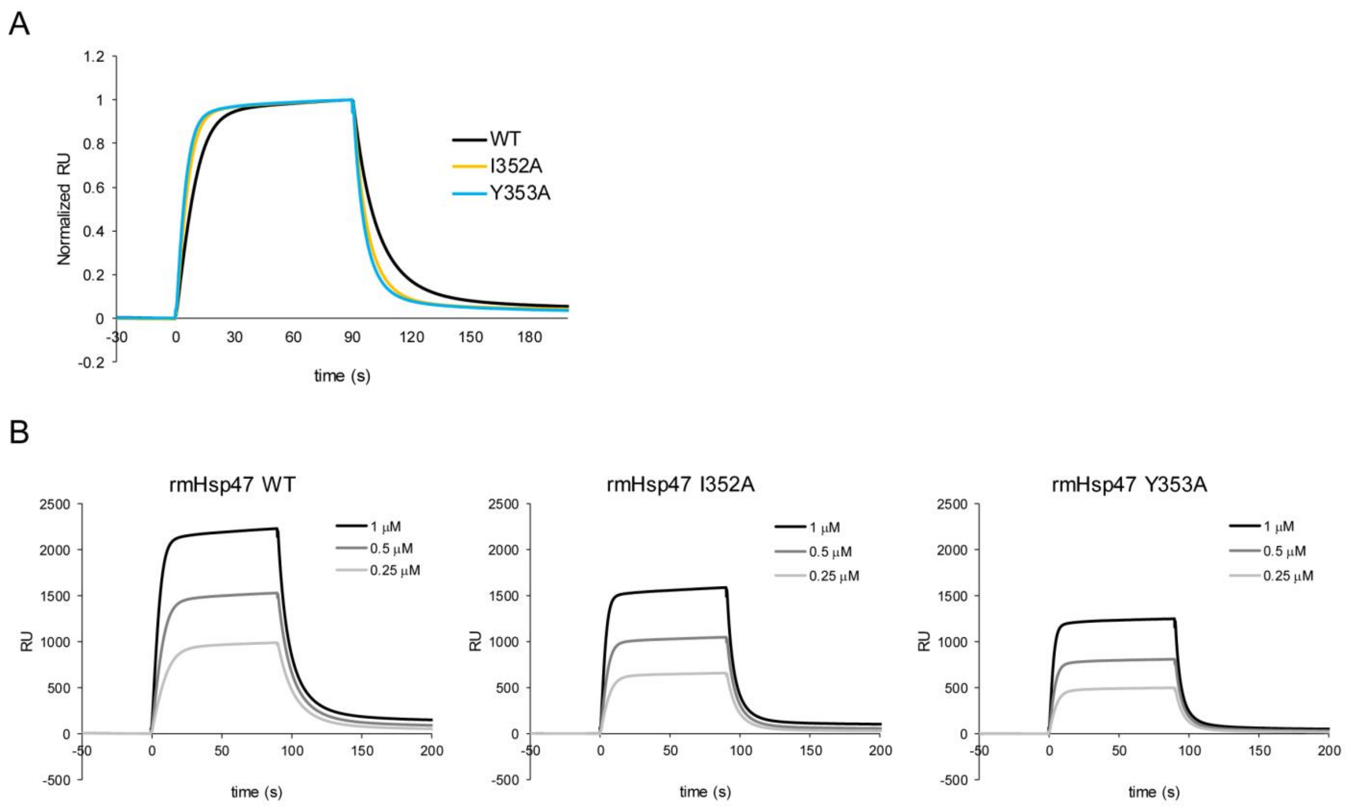

Figure S3. Sensor grams on surface plasmon resonance (SPR) detecting the binding of recombinant mouse Hsp47 (rmHsp47) with collagen

(A) Sensor grams are shown as normalized curve adjusted at first point on dissociation Hsp47 from collagen immobilized sensor tip. I352A and Y353A of rmHsp47 dissociate faster than wild type rmHsp47. (B) The concentration dependences of the binding kinetics. The dissociation kinetics (KD) were determined using the fitting software, Anabel.
